# Selective in vitro Synergistic Evaluation of Probiotic Tolerant morpholinyl- and 4-ethylpiperazinyl-Imidazole-chalcone Derivatives on Gastrointestinal System Pathogens

**DOI:** 10.1007/s00284-024-03788-5

**Published:** 2024-07-03

**Authors:** Tuncay Söylemez, Zafer Asım Kaplancıklı, Derya Osmaniye, Yusuf Özkay, Fatih Demirci

**Affiliations:** 1 Institut Für Lebensmittelchemie, Gottfried Wilhelm Leibniz Universität Hannover, Callinstraße 5, 30167 Hannover, Germany; 2 Faculty of Pharmacy, Department of Pharmaceutical Chemistry, Anadolu University, Eskişehir, Türkiye; 3 Faculty of Pharmacy, Pharmacognosy Department, Anadolu University, Eskişehir, Türkiye; 4 Faculty of Pharmacy, Eastern Mediterranean University, Famagusta, N. Cyprus Cyprus

## Abstract

Imidazole-chalcone compounds are recognised for their broad-spectrum antimicrobial properties. Probiotic-friendly, selective new-generation antimicrobials prove to be more efficient in combating gastrointestinal system pathogens. The aim of this study is to identify imidazole-chalcone derivatives that probiotics tolerate and evaluate their in vitro synergistic antimicrobial effects on pathogens. In this study, fifteen previously identified imidazole-chalcone derivatives were analyzed for their in vitro antimicrobial properties against gastrointestinal microorganisms. Initially, the antimicrobial activity of pathogens was measured using the agar well diffusion method, while the susceptibility of probiotics was determined by microdilution. The chosen imidazole-chalcone derivatives were assessed for synergistic effects using the checkerboard method. Four imidazole-chalcone derivatives to which probiotic bacteria were tolerant exhibited antibacterial and antifungal activity against the human pathogens tested. To our knowledge, this study is the first to reveal the fractional inhibitory concentration (FIC) of combinations of imidazole-chalcone derivatives. Indeed, the minimum inhibitory concentrations (MIC) for morpholinyl- (ZDO-3f) and 4-ethylpiperazinyl- (ZDO-3 m) imidazole-chalcones were notably low when tested against *E. coli* and *B. subtilis*, with values of 31.25 μg/mL and 125 μg/mL, respectively. The combination of morpholinyl- and 4-ethylpiperazinyl derivatives demonstrated an indifferent effect against *E. coli*, but an additive effect was observed for *B. subtilis*. Additionally, it was observed that imidazole-chalcone derivatives did not exhibit any inhibitory effects on probiotic organisms like *Lactobacillus fermentum* (CECT-5716), *Lactobacillus rhamnosus* (GG), and *Lactobacillus casei* (RSSK-591). This study demonstrates that imidazole-chalcone derivatives that are well tolerated by probiotics can potentially exert a synergistic effect against gastrointestinal system pathogens.

**Supplementary Information:**

The online version contains supplementary material available at 10.1007/s00284-024-03788-5.

## Introduction

The imidazole ring consists of a five-membered ring structure containing a hydrogen-binding domain and an electron-donor nitrogen system. The first imidazole was described by Fischer in 1882, but the nature of the ring system was elucidated by Freud and Kuhn in 1890. Imidazoles are significant due to their biological activities among their isomers. In particular, imidazole exhibits a wide range of biological activities, including antimicrobial, antituberculosis, antioxidant, anti-inflammatory, anticonvulsant, antidepressant-anxiolytic, antihypertensive, anticancer, and antifungal properties [1].

Imidazole derivatives, including compounds like histamine, biotin, alkaloids, and nucleic acids, as well as their derivatives, continue to play a crucial role in the fields of medical and pharmaceutical chemistry. The design and synthesis of imidazole derivatives are primarily focused on their potential applications in antibacterial, anticancer, antifungal, analgesic, and anti-HIV activities [2]. In recent times, imidazole derivatives have been investigated for their antimicrobial properties and their potential utility as therapeutic agents [3, 4]. Over the past few decades, the synthesis and design of hybrid compounds containing imidazole have been demonstrated to possess pharmacological activity [5]. It is well established that chalcones exhibit remarkable antimicrobial activity [6, 7].

**Fig. 1:**
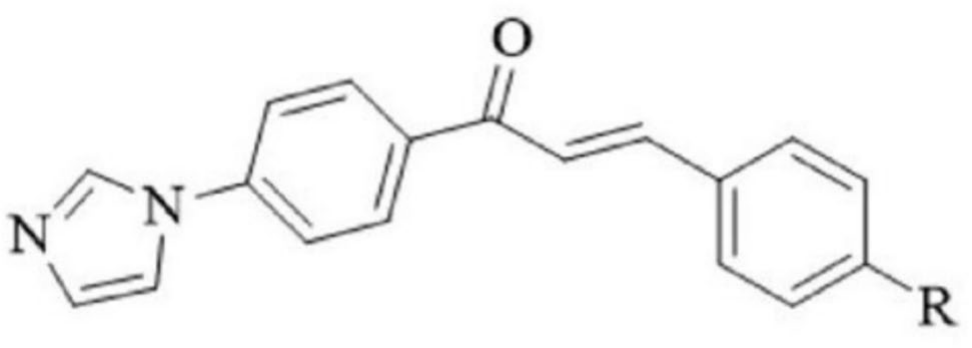
Chemical structures of the imidazole-chalcone compounds (1–15). This figure displays the chemical structures of fifteen different imidazole-chalcone compounds labeled as 1 to 15. Each structure includes annotations of relevant functional groups and any significant structural features

In the context of our imidazole research projects, we have previously designed and synthesized 15 imidazole derivatives, incorporating chalcone pharmacophores as potential antifungal components, as depicted in Fig. 1 [8]. The activity studies demonstrated the effectiveness of certain compounds as antifungal agents.

It is widely recognised that the mucosa of the gastrointestinal system is the largest exposed membrane in the body. This membrane allows for efficient absorption of nutrients but also exposes the body to potential external toxins and pathogens [9]. The gastrointestinal system microbiota comprises both pathogens and probiotics [10].

In recent decades, the use of probiotics has become more common due to their beneficial effects on the health of their hosts. They are often referred to as bacterial therapeutics, microbial therapeutics, or regulators of the bacterial immune system, and their clinical effects are studied in greater detail [11].

The intestinal microflora comprises a diverse ecosystem with more than 400 bacterial species, found in both the lumen and attached to the mucosa without penetrating the bowel wall [12]. These bacteria are crucial for the enterohepatic circulation process, where liver-conjugated metabolites are deconjugated in the intestine by bacterial enzymes. This process aids in the absorption of metabolites across the mucosa and their return to the liver via the portal circulation [13]. Disruption of this flora by antibiotics can affect the excretion and blood levels of various compounds. Additionally, the microflora aids in fibre digestion, synthesises certain vitamins, and can prevent infections by interfering with pathogens [14]. However, disturbances in the normal flora balance can lead to infections by external pathogens and the overgrowth of internal pathogens such as *Clostridium difficile* [12].

Many studies and approaches targeting gastrointestinal tract pathogens have often overlooked the presence of probiotics. It is challenging to identify a specific and targeted antibiotic or antibacterial compound in the diverse bacterial species of the gastrointestinal flora, which comprises hundreds of species.

Antibiotic treatment can alter the gut bacterial microbiota both quantitatively and qualitatively, resulting in a reduction or loss of certain species [15].

The primary objective of this research project was to explore the synergistic potential of fifteen imidazole-chalcone derivatives. Our aim was to identify compounds that exhibit selective antimicrobial effects against gastrointestinal pathogens while sparing beneficial microorganisms within the microbiome. We hypothesize that these imidazole-chalcone derivatives will not adversely affect beneficial microbiota but will demonstrate activity against harmful pathogens. Our next step is to evaluate the synergistic effects among these derivatives. This approach aims to select a combination of two derivatives that are less harmful and offer greater benefits for the intestinal system and its flora. To the best of our knowledge, this specific exploration of selective antimicrobial effects and their impact on the microbiome has not been previously documented.

## Material and Methods

### Test Substances

**Fig. 2:**
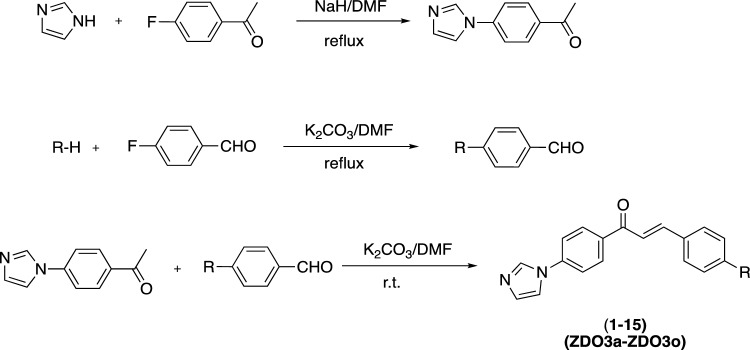
Synthesis route of the test compounds (ZDO3a-ZDO3o). This figure illustrates the step-by-step synthetic pathway used to produce the test compounds labeled ZDO3a to ZDO3o. Key reagents, reaction conditions, and intermediates are clearly marked at each stage of the synthesis

Fifteen imidazole-chalcone derivatives were synthesised, and their structures are shown in Fig. 2. These compounds were provided by the Department of Pharmaceutical Chemistry, Faculty of Pharmacy, Anadolu University, as described in detail in Table 1 [8]. The antimicrobial agents, including levofloxacin for *Clostridium difficile* (ATCC 9689), ampicillin, and amphotericin B for yeast, were procured from Sigma–Aldrich (catalogue numbers: 28266, A9518, A4888). All other chemical consumables used in the study were of microbiological or biochemical purity, unless otherwise specified.

**Table 1:** Imidazole-chalcone derivative synthesis yields and melting points [8]

Compound	Code	R-functional group	Yield (%)	Melting point ^o^C
1	ZDO-3a	4-methylphenoxy	79	146–148
2	ZDO-3b	4-methylphenylthio	84	168–169
3	ZDO-3c	4-methoxyphenoxy	92	168–170
4	ZDO-3d	4-methoxyphenylthio	80	177–179
5	ZDO-3e	Pyrrolidinyl	77	186–188
6	ZDO-3f	Morpholinyl	79	214–215
7	ZDO-3 g	Piperidinyl	73	198–199
8	ZDO-3 h	3-methylpiperidinyl	80	163–165
9	ZDO-3i	4-methylpiperidinyl	77	189–191
10	ZDO-3j	3,5-dimethylpiperidinyl	81	160–161
11	ZDO-3 k	4-benzylpiperidinyl	83	178–180
12	ZDO-3 l	4-methylpiperazinyl	82	205–207
13	ZDO-3 m	4-ethylpiperazinyl	86	188–189
14	ZDO-3n	4-(2-dimethylaminoethyl)piperazinyl	78	146–149
15	ZDO-3o	4-(3-dimethylaminopropyl)piperazinyl	79	138–141

### Microorganism

The bacteria obtained from culture collections and used in this study were *Lactobacillus fermentum* (CECT-5716), *Lactobacillus rhamnosus* (GG), *Lactobacillus casei* (RSSK-591), *Escherichia coli* (NRRL B-3008), *B. subtilis*, *Clostridium difficile* (ATCC 9689), and the yeasts *Candida albicans* (ATCC 10231) and *Candida krusei* (ATCC 6258), respectively.

### Microorganism Cultivation

MRS (MAN, ROGOSA, and SHARPE, Merck, Darmstadt, Germany) agar solid medium and smear plaque were prepared and utilised for the cultivation of *Lactobacillus spp.* MHA (Mueller Hinton Agar, Merck, Darmstadt, Germany) was used for *E. coli*, *B. subtilis*, and *C. difficile*, and PDA (Potato Dextrose Agar, Merck, Darmstadt, Germany) was freshly prepared and employed for the cultivation of *C. albicans* and *C. krusei*.

### Agar Well Diffusion is Used For Antimicrobial Activity Evaluation of Pathogens

This study was designed to assess the selective antimicrobial activity of imidazole-chalcone derivatives against gastrointestinal system pathogens [16]. Initially, all compounds were tested at a concentration of 2000 µg/mL. Fresh cultures of *E. coli*, *B. subtilis*, *C. difficile*, *C. albicans*, and *C. krusei* were incubated for 24 h. The turbidity of the suspension was adjusted using a densitometer (Bioland; ATC) to match that of a 0.5 McFarland standard. Inoculums were prepared from 24 h yeast cultures on PDA and bacteria from 24 h cultures on Mueller Hinton Agar. Then, 100 µL of each microorganism sample was transferred.

Six-millimetre-diameter wells were created on the solidified agar medium, and 20 µL of each test sample was placed in the wells. The presence of an inhibition zone indicated activity. After 24 h of incubation at 37 °C, the resulting inhibition zones (IZ) were measured. Ampicillin, Levofloxacin, and Amphotericin B were used as standards for comparison and control. The experiment was conducted in duplicate, and the results were reported as the mean of the measurements.

### Antimicrobial Susceptibility Test for Probiotics

The susceptibility of probiotic bacteria in the gastrointestinal system to imidazole-chalcone derivatives with antimicrobial activity was assessed using the Agar Well Diffusion method [17]. The concentration of imidazole-chalcone was set at 2000 µg/mL. *L. fermentum*, *L. rhamnosus*, and *L. casei* species were selected, and fresh cultures that had been incubated for 24 h were used. The turbidity of the suspension was adjusted using a densitometer to match that of a 0.5 McFarland standard. Microorganisms were inoculated onto MRS (MAN, ROGOSA, and SHARPE) agar plates using the smear plaque method.

Six-millimetre-diameter wells were created in the agar, and 20 µL of the test samples were placed into each well. The presence of an inhibition zone indicated activity. After 24 h of incubation at 37 °C, the resulting inhibition zones (IZ) were measured. Ampicillin was used as a reference for comparison and control. The experiments were conducted in duplicate.

### Microdilution Test for Antimicrobial Susceptibility

Broth microdilutions were performed in strict accordance with the Clinical and Laboratory Standards Institute (CLSI) protocol [18]. The setup included one vertical row for growth control, another for antibiotic control, and vertical wells used for different concentrations of imidazole-chalcone derivatives. The first well contained 1000 µg/mL and 200 µL of imidazole-chalcone derivatives, while the other nine wells in the same row were filled with 100 µL of mueller hinton broth (MHB). A separate sterile pipette was employed to transfer 100 μL of the mixture from the first well into the second well and thoroughly mix it. This serial dilution process was repeated up to the tenth well, with 100 μL transferred from one well to the next. Finally, 100 μL was removed from the tenth well and discarded. This process established a concentration range of 1000–1.95 µg/mL for the four selected imidazole-chalcone derivatives.

*E. coli*, *B. subtilis*, *C. albicans*, and *C. krusei* were the target microorganisms, and fresh cultures that had been incubated for 24 h were used. Cell density settings were adjusted using a cell densitometer, and 10 μL of microorganisms, diluted to a 0.5 McFarland setting (approximately 5 × 105 CFU/mL), were added to the wells of the microtitration plates. The final well volume was 200 μL. After a 24 h incubation at 37 °C, 20 μL of a 0.01% (w/v) resazurin solution was added to the wells and incubated for an additional 2 h at 37 °C. The results were reported as MIC (μg/mL) values.

For comparison, positive and negative controls were used. Ampicillin served as an antibacterial agent, and Amphotericin B was used as an antifungal agent [19, 20]. The experiments were conducted in triplicate.

### Checkerboard Test

Broth microdilutions were performed in strict accordance with the Clinical and Laboratory Standards Institute (CLSI) protocol [18]. The setup included one vertical row for growth control, another for antibiotic control, and vertical wells used for different concentrations of imidazole-chalcone derivatives. The first well contained 1000 µg/mL and 200 µL of imidazole-chalcone derivatives, while the other nine wells in the same row were filled with 100 µL of mueller hinton broth (MHB). A separate sterile pipette was employed to transfer 100 μL of the mixture from the first well into the second well and thoroughly mix it. This serial dilution process was repeated up to the tenth well, with 100 μL transferred from one well to the next. Finally, 100 μL was removed from the tenth well and discarded. This process established a concentration range of 1000–1.95 µg/mL for the four selected imidazole-chalcone derivatives.

*E. coli*, *B. subtilis*, *C. albicans*, and *C. krusei* were the target microorganisms, and fresh cultures that had been incubated for 24 h were used. Cell density settings were adjusted using a cell densitometer, and 10 μL of microorganisms, diluted to a 0.5 McFarland setting (approximately 5 × 105 CFU/mL), were added to the wells of the microtitration plates. The final well volume was 200 μL. After a 24 h incubation at 37 °C, 20 μL of a 0.01% (w/v) resazurin solution was added to the wells and incubated for an additional 2 h at 37 °C. The results were reported as MIC (μg/mL) values.

For comparison, positive and negative controls were used. Ampicillin served as an antibacterial agent, and Amphotericin B was used as an antifungal agent [19, 20]. The experiments were conducted in triplicate [21]. Positive and negative controls were included to assess the validity of the experiments. Ampicillin was utilised as the antibacterial control.

The fractional ınhibitory concentration Index (FICI) was calculated as follows: FIC ≤ 0.5: Synergism; FIC 0.5–1: Additive; FIC 1–4: Indifferent; FIC > 4: Antagonistic Effect [22, 23].

1 $$\sum {\text{FIC}}\, = \,{\text{FIC A}}\, + \,{\text{FIC B}}\, = \,\left[ {\text{A}} \right]/{\text{MICA}}\, + \,\left[ {\text{B}} \right]/{\text{MICB}}$$

where:FIC A is the MIC of sample A in the combination divided by the MIC of sample A alone. FIC B is the MIC of sample B in the combination divided by the MIC of sample B alone.

## Results

### Antimicrobial Activity

**Fig. 3:**
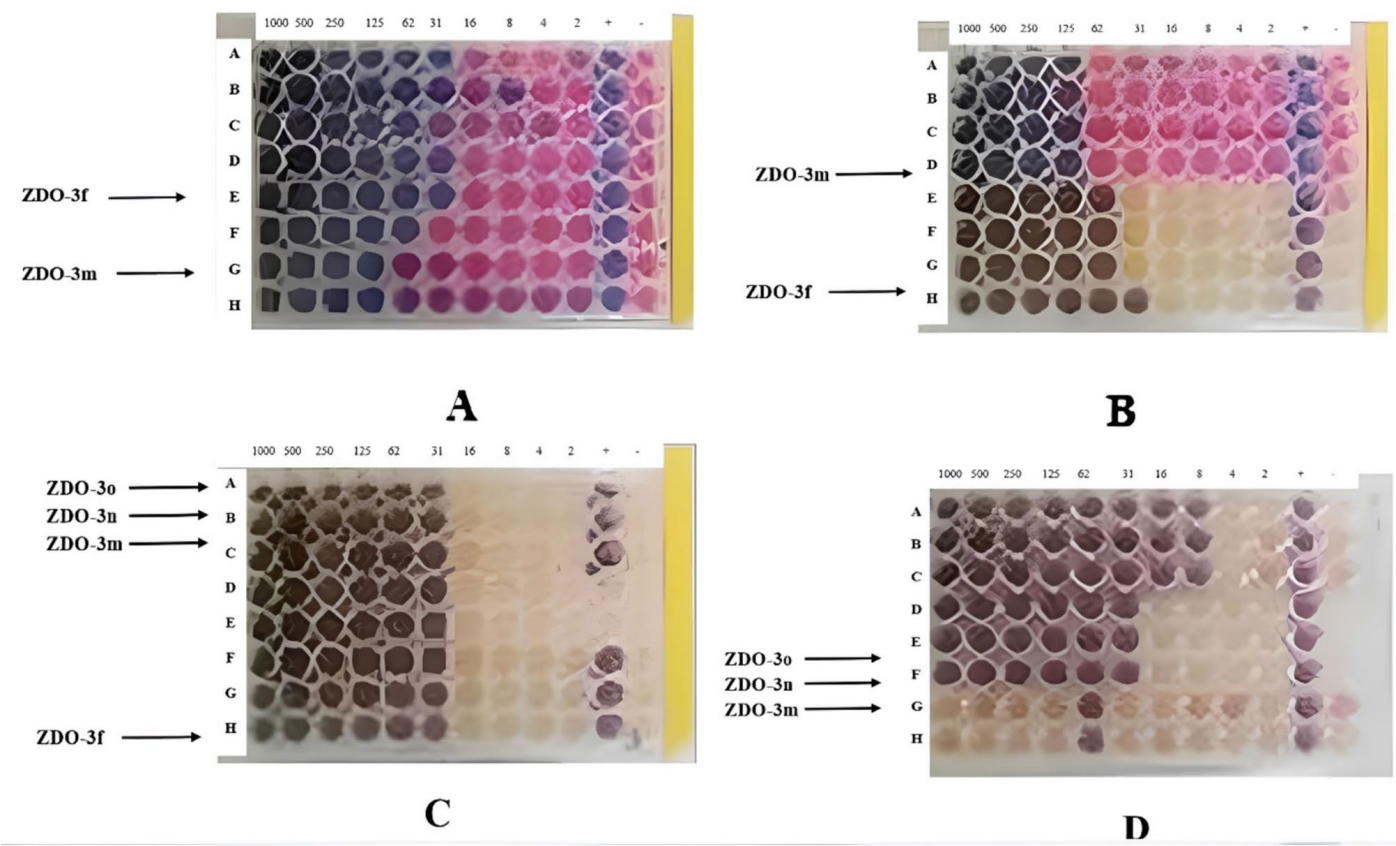
Minimum inhibitory concentration (MIC) values of imidazole-chalcone derivatives (ZDO-3f to ZDO-3o) against gastrointestinal pathogens. It presents the MIC values of imidazole-chalcone derivatives (labeled among ZDO-3f to ZDO-3o) against four gastrointestinal pathogens across a concentration range of 2 to 1000 µg/mL. First purple coloring indicates MIC values. The pathogens tested include: **a** *Escherichia coli *(*E. coli*) **b** *Bacillus subtilis *(*B. subtilis*) **c** *Candida albicans *(*C. albicans*) **d** *Candida krusei *(*C. krusei*)

Among the fifteen imidazole-chalcone derivatives, four exhibited strong antimicrobial activity against gastrointestinal pathogens. The agar well diffusion method showed clear inhibition zones for these derivatives (Table 2). MIC values were determined for the active compounds, revealing significant inhibitory effects at low concentrations (Table 3, Fig. 3). The minimum inhibitory concentration (MIC) values for the compounds were determined as follows: ZDO-3f exhibited an MIC of 31 µg/mL against both *E. coli* and *B. subtilis*. For ZDO-3 m, the MIC was 125 µg/mL against *E. coli* and *B. subtilis*, and 62 µg/mL against *C. krusei*. The MIC for ZDO-3n was 31 µg/mL against *C. krusei*. Similarly, ZDO-3o demonstrated an MIC of 31 µg/mL against both *C. albicans* and *C. krusei*.

**Table 2:** Results of agar diffusion method against pathogens

Samples	Inhibition zone diameter (mm)				
	*E. coli*	*B. subtilis*	*C. difficile*	*C. albicans*	*C. krusei*
ZDO-3f	9	8	0	0	0
ZDO-3 m	5	6	0	0	2
ZDO-3n	0	0	0	0	2
ZDO-3o	0	0	0	3	3
Ampicillin	8	15	**–**	**–**	–
Amoxicillin B	**–**	**–**	**–**	7	5
levofloxacin	**–**	**–**	12	**–**	**–**

-: not tested

**Table 3:** Minimum ınhibition concentrations of ımidazole-chalcone derivatives

	MIC values (µg/mL)			
	*E.coli*	*B. subtilis*	*C. albicans*	*C. krusei*
Sample Code				
ZDO-3f	31	31	–	–
ZDO-3 m	125	125	–	62
ZDO-3n	–	–	–	31
ZDO-3o	–	–	31	31
Ampicillin	250	4	–	–
Amphotericin B	–	–	8	31

### Probiotic Tolerance

The selected imidazole-chalcone derivatives did not inhibit the growth of probiotic bacteria (*L. fermentum*, *L. rhamnosus*, *L. casei*) when tested at 2000 μg/mL, unlike the control antibiotic Ampicillin (Fig. S1).

### Checkerboard Assay

**Fig. 4:**
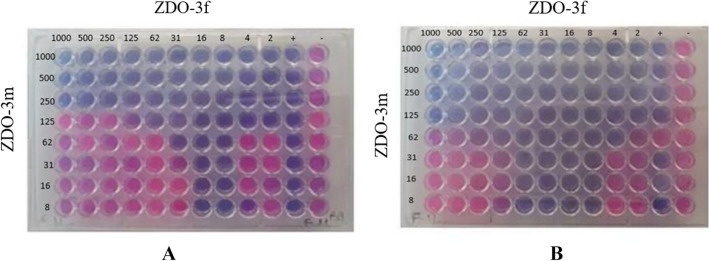
Fractional Inhibitory Concentration Index (FICI) values affected by mixing different concentrations of imidazole chalcone derivatives. Mixtures in different combinations were created in each microwell by adding ZDO-3f in the range of 2 to 1000 µg/mL in the column and ZDO-3m in the range of 8 to 1000 µg/mL in the row. The first purple color, where both derivatives were at the lowest concentrations, was taken into account. Pathogens tested include: **a** *Bacillus subtilis *(*B. subtilis*) **b** *Escherichia coli (E. coli)*

The combination of morpholinyl- and 4-ethylpiperazinyl derivatives exhibited an additive effect against *B. subtilis*, with FICI values indicating no antagonistic interactions (Table 4, Fig. 4).

**Table 4:** Synergistic effect of ımidazole-chalcone derivatives

	*ΣFIC* value (µg/mL)			
Microorganisms	Ampicillin	Combination	*ΣFIC*	Results
*E. coli*	250	ZDO-3f + ZDO-3 m	2125	Indiffirent
*B. subtilis*	4	ZDO-3f + ZDO-3 m	750	Additive

In the present study, the microbiological interactions of fifteen different known imidazole-chalcone derivatives were investigated. Besides determining the minimum ınhibitory concentration (MIC), the fractional ınhibitory concentration ındex (FICI) was calculated using the checkerboard test for combination studies.

The in vitro antimicrobial activity of these fifteen imidazole-chalcone derivatives was assessed against five different gastrointestinal system pathogens, including both bacteria and yeasts. Initially, four of these imidazole-chalcone derivatives exhibited strong antimicrobial activity against the tested pathogens, as indicated in Table 2. Subsequently, these selected imidazole-chalcones were further evaluated for their antimicrobial effects against non-pathogenic microorganisms, and they displayed relatively high antimicrobial activity.

To initially determine the in vitro antimicrobial activity of the imidazole-chalcone derivatives against gastrointestinal system pathogens, the agar well diffusion method was employed, and this activity was assessed and quantified using zone diameters (as depicted in Fig. S2). These experiments were conducted in triplicate, and the reported values represent the averages of the results.

The efficacy of four distinct imidazole-chalcone derivatives, which had previously shown susceptibility against gastrointestinal system pathogens, was re-evaluated in vitro against probiotic bacteria using the agar well diffusion method [16]. The results demonstrated that there was no susceptibility observed for probiotic bacteria when exposed to imidazole-chalcone derivatives, in contrast to the standard Ampicillin (with zones of inhibition typically ranging from 9 to 12 cm). These findings are illustrated in Figure S1.

The imidazole-chalcone derivatives demonstrated antimicrobial activity against certain tested gastrointestinal pathogens (Table 2). However, they did not exhibit antibacterial activity against the three probiotic bacteria tested in our study (Fig. S1). This preliminary data suggests that these derivatives may have a degree of selectivity in their antimicrobial effects, though further investigation is needed to confirm these findings.

The assays [7] were conducted to determine the minimum inhibitory concentration (MIC) of imidazole-chalcone derivatives against the pathogens listed in Table 3. Figure 3 illustrates the MIC test results for each pathogen: *E. coli* (Fig. 3A), *B. subtilis* (Fig. 3B), *C. albicans* (Fig. 3C), and *C. krusei* (Fig. 3D). Each part of Fig. 3 (A, B, C, and D) refers to separate panels within the figure, each representing the MIC tests for a specific microorganism. These visualizations provide additional insights to complement the data presented in Table 3.

The checkerboard test [7] was performed to evaluate the effects of various concentrations of imidazole-chalcone derivatives on the test microorganisms. The fractional inhibitory concentration (FIC) was calculated to determine the nature of the interactions (synergistic, additive, indifferent, or antagonistic), with the results presented in Table 4. Specifically, the MIC values for the tested combinations and their effects on *E. coli* and *B. subtilis* are shown. For *E. coli*, the combination of ZDO-3f and ZDO-3 m resulted in a ΣFIC value of 2.125, indicating an indifferent effect. For *B. subtilis*, the combination of ZDO-3f and ZDO-3 m resulted in a ΣFIC value of 0.75, indicating an additive effect. These interactions are visualized in Fig. 4, which depicts the checkerboard results and provides a detailed view of the interactions between imidazole-chalcone derivatives and the microorganisms.

## Discussion

To the best of our knowledge, this study is the first to report the additive effects of imidazole derivatives against the selected microorganisms. Previous research by Dupont and Drouhet [19] investigated the synergistic effect of imidazole and Amphotericin B combinations against Candida spp. but found a less pronounced antimicrobial effect when the two agents were tested together [19]. It has been documented that imidazole derivatives generally have fewer side effects compared to Amphotericin B, suggesting a more favorable safety profile in certain therapeutic applications [25].

The antimicrobial action of imidazole-chalcone derivatives is primarily associated with the reactive enone moiety, which acts as a Michael reaction acceptor, binding to thiol groups on specific proteins. Many chalcones, including these imidazole-chalcone derivatives, are recognized for their ability to inhibit the biosynthesis of cell walls in pathogenic Candida species, thus contributing to their antimicrobial potential [7]. This aligns with findings by Gupta et al. [26], who demonstrated the efficacy of chalcone derivatives in targeting fungal cell wall synthesis [26]. Compared to other antimicrobial agents, the mechanism of action involving the enone moiety may offer a distinct advantage in targeting specific pathogens without affecting probiotics.

In our current study, the combination of the selected imidazole-chalcones (1–4) exhibited an additive effect, indicating their potential utility against gastrointestinal system pathogens. Importantly, these compounds did not show activity against the tested human probiotic microorganisms, suggesting a favorable safety profile. Similar studies, such as that by Belkaid et al. [27], have highlighted the importance of selective antimicrobial activity, particularly in preserving beneficial microbiota while targeting pathogens [27].

It is well acknowledged that in vitro studies conducted under laboratory conditions may not always yield the same results in vivo. Therefore, obtaining more reliable results through subsequent in vivo experiments is essential. This necessity is supported by the work of Hirsch et al. [28], which emphasized the discrepancies between in vitro and in vivo antimicrobial activities and the need for comprehensive in vivo assessments [28]. This underscores the importance of validating our in vitro findings with animal models or clinical trials to ensure translational efficacy.

The results of three repeated processes at different times and under the same conditions were analyzed using standard deviation calculations, with no statistically non-significant values observed. This methodological rigor aligns with standards set by prior research, ensuring the reliability of our findings [29].

While this study focuses on a subset of fifteen imidazole-chalcone derivatives and specific probiotics and pathogens relevant to the gastrointestinal microbiota, it recognizes the broader unexplored landscape. Discovering that four compounds among the derivatives exhibit antimicrobial effects against pathogens while remaining insensitive to probiotics is a promising outcome. This suggests potential utility in targeted antimicrobial applications. However, broader screenings with a more extensive array of derivatives and microbial strains are warranted for a comprehensive understanding. The need for broader screenings is further underscored by recent reviews on antimicrobial resistance, which call for expansive and diverse antimicrobial testing to combat emerging resistant strains [30].

This study presents a focused snapshot of antimicrobial activities and synergies within a specific context, guiding further exploration and refinement in antimicrobial research and strategies. The findings here contribute to the growing body of literature advocating for the development of selective antimicrobial agents, as highlighted in recent studies by Nematollahi et al. [31] and Mezbege et al. [32], which emphasize precision in antimicrobial therapy to minimize resistance development [31, 32]. Future research should explore the clinical implications of these findings, particularly how they might influence current treatment protocols or contribute to the development of new, more effective therapies.

## Conclusion

In conclusion, certain imidazole-chalcone derivatives demonstrate a distinctive characteristic by affecting pathogens while sparing probiotic organisms. Furthermore, the utilization of synergistic compounds may reduce the likelihood of resistance development among target microorganisms, offering additional advantages for potential applications.

We aimed to investigate solutions for these three problems simultaneously. Firstly, we sought to obtain an antimicrobial agent effective against gastrointestinal system pathogens. Secondly, this antimicrobial activity should not harm the presence and effectiveness of probiotics. Finally, using the checkerboard method for dual combinations, we aimed to achieve more effective results with fewer antimicrobial substances overall, thereby reducing side effects.

To the best of our knowledge, this study is the first to report the additive effects of imidazole derivatives against the selected microorganisms. Previous research by Dupont and Drouhet (1979) investigated the synergistic effect of imidazole and Amphotericin B combinations against Candida spp. but found a less pronounced antimicrobial effect when the two agents were tested together [19]. It has been documented that imidazole derivatives generally have fewer side effects compared to Amphotericin B, suggesting a more favorable safety profile in certain therapeutic applications [25].

Moreover, our utilization of the checkerboard method aligns with recent advancements in antimicrobial research, such as the three-dimensional synergy analysis conducted by Stein et al. [33], which evaluated the benefits of triple antibiotic combinations against multidrug-resistant *Klebsiella pneumoniae* strains. While their study focused on determining the efficacy of colistin-based combinations against carbapenem-non-susceptible *K. pneumoniae*, our findings complement their work by elucidating the nuanced interactions within antimicrobial compounds, particularly in the context of gastrointestinal system pathogens [33].

While Ballan et al. [34] highlighted concerns about imidazole propionate’s impact on insulin signalling, their research showed that probiotics could counteract this effect. This finding aligns well with our study’s investigation into the compatibility of probiotics with imidazole-chalcone derivatives [34].

Our expectation from this study is to demonstrate that the majority of probiotics in the gastrointestinal microbiota are tolerant to these imidazole-chalcone derivatives in future research. The study aims to clearly demonstrate “synergistic” effects rather than merely "additive effects" against some pathogens. Thus, it will illuminate studies addressing gastrointestinal system pathogens, serving as both a methodological approach and elucidating the role of synergistic factors.

### Supplementary Information

Below is the link to the electronic supplementary material.

Supplementary file1 (DOCX 212 kb)

Supplementary file2 (DOCX 1703 kb)

## References

[1] Shallal MAH (2019) A literature review on the imidazole. Am Int J Multidiscip Sci Res 5(1):1–11. 10.46281/aijmsr.v5i1.231 10.46281/aijmsr.v5i1.231

[2] Verma A, Joshi S, Singh D (2013) Imidazole: having versatile biological activities. J Chem 2013:12. 10.1155/2013/329412 10.1155/2013/329412

[3] Sharma D, Narasimhan B, Kumar P et al (2009) Synthesis, antimicrobial and antiviral evaluation of substituted imidazole derivatives. Eur J Med Chem 44(6):2347–2353. 10.1016/j.ejmech.2008.08.010 18851889 10.1016/j.ejmech.2008.08.010

[4] Rani N, Sharma A, Singh R (2013) Imidazoles as promising scaffolds for antibacterial activity: a review. Mini Rev Med Chem 13(12):1812–1835. 10.2174/13895575113136660091 24032508 10.2174/13895575113136660091

[5] Kouznetsov VV, Gómez-Barrio A (2009) Recent developments in the design and synthesis of hybrid molecules based on aminoquinoline ring and their antiplasmodial evaluation. Eur J Med Chem 44(8):3091–3113. 10.1016/j.ejmech.2009.02.024 19361896 10.1016/j.ejmech.2009.02.024

[6] Tratrat C, Haroun M, Xenikakis I, Liaras K, Tsolaki E, Eleftheriou P et al (2019) Design, synthesis, evaluation of antimicrobial activity and docking studies of new thiazole-based chalcones. Curr Top Med Chem 19(5):356–375 30706816 10.2174/1568026619666190129121933

[7] Hellewell L, Bhakta S (2020) Chalcones, stilbenes and ketones have anti-infective properties via inhibition of bacterial drug-efflux and consequential synergism with antimicrobial agents. Access Microbiol. 10.1099/acmi.0.000105 33005869 10.1099/acmi.0.000105 PMC7523622

[8] Çavuşoğlu BK, Sağlık BN, Osmaniye D, Levent S, Acar Çevik U, Karaduman AB, Özkay Y, Kaplancıklı ZA (2017) Synthesis and biological evaluation of new thiosemicarbazone derivative schiff bases as monoamine oxidase inhibitory agents. Molecules 23(1):60. 10.3390/molecules23010060 29283399 10.3390/molecules23010060 PMC6017703

[9] Sekirov I, Russell SL, Antunes LC, Finlay BB (2010) Gut microbiota in health and disease. Physiol Rev 90(3):859–904. 10.1152/physrev.00045.2009 20664075 10.1152/physrev.00045.2009

[10] Chermesh I, Eliakim R (2006) Probiotics and the gastrointestinal tract: where are we in 2005? World J Gastroenterol 12(6):853–857. 10.3748/wjg.v12.i6.853 16521211 10.3748/wjg.v12.i6.853 PMC4066148

[11] Kerry RG, Patra JK, Gouda S, Park Y, Shin HS, Das G (2018) Benefaction of probiotics for human health: A review. J Food Drug Anal 26(3):927–939. 10.1016/j.jfda.2018.01.002 29976412 10.1016/j.jfda.2018.01.002 PMC9303019

[12] Gorbach SL (1996) Microbiology of the gastrointestinal tract. In: Baron S (ed) Medical Microbiology, 4th edn. Galveston, TX 21413258

[13] Durník R, Šindlerová L, Babica P, Jurček O (2022) Bile acids transporters of enterohepatic circulation for targeted drug delivery. Molecules 27(9):2961. 10.3390/molecules27092961 35566302 10.3390/molecules27092961 PMC9103499

[14] Hou K, Wu ZX, Chen XY, Wang JQ, Zhang D, Xiao C, Zhu D, Koya JB, Wei L, Li J, Chen ZS (2022) Microbiota in health and diseases. Sig Transduct Target Ther 7:135. 10.1038/s41392-022-00974-4 10.1038/s41392-022-00974-4 PMC9034083 35461318

[15] Éliás AJ, Barna V, Patoni C, Demeter D, Veres DS, Bunduc S, Erőss B, Hegyi P, Földvári-Nagy L, Lenti K (2023) Probiotic supplementation during antibiotic treatment is unjustified in maintaining the gut microbiome diversity: a systematic review and meta-analysis. BMC Med 21(1):262. 10.1186/s12916-023-02961-0 37468916 10.1186/s12916-023-02961-0 PMC10355080

[16] Magaldi S, Mata-Essayag S, Hartung de Capriles C, Perez C, Colella MT, Olaizola C, Ontiveros Y (2004) Well diffusion for antifungal susceptibility testing. Int J Infect Dis 8(1):39–45. 10.1016/j.ijid.2003.03.002 14690779 10.1016/j.ijid.2003.03.002

[17] Tagg JR, McGiven AR (1971) Assay system for bacteriocins. Appl Microbiol 21(5):943. 10.1128/am.21.5.943-943.1971 4930039 10.1128/am.21.5.943-943.1971 PMC377313

[18] Wayne PA (2012) Clinical and Laboratory Standards Institute. In: Megan P, Larrisey MA (eds) Approved standard. Wayne, Pennsylvania, pp 1–88

[19] Dupont B, Drouhet E (1979) In vitro synergy and antagonism of antifungal agents against yeast-like fungi. Postgrad Med J 55(647):683–686. 10.1136/pgmj.55.647.683 523360 10.1136/pgmj.55.647.683 PMC2425645

[20] Villalba MI (2020) *Estudios a micro y nanoescala de la respuesta de Bordetella pertussis a condiciones del entorno* (Doctoral dissertation). Universidad Nacional de La Plata, Facultad de Ciencias Exactas, Departamento de Ciencias Biológicas

[21] Bajaksouzian S, Visalli MA, Jacobs MR, Appelbaum PC (1997) Activities of levofloxacin, ofloxacin, and ciprofloxacin, alone and in combination with amikacin, against *Acinetobacters* as determined by checkerboard and time-kill studies. Antimicrob Agents Chemother 41(5):1073–1076. 10.1128/AAC.41.5.1073 9145872 10.1128/AAC.41.5.1073 PMC163853

[22] Meletiadis J, Pournaras S, Roilides E, Walsh TJ (2010) Defining fractional inhibitory concentration index cutoffs for additive interactions based on self-drug additive combinations, Monte Carlo simulation analysis, and in vitro-in vivo correlation data for antifungal drug combinations against *Aspergillus fumigatus*. Antimicrob Agents Chemother 54(2):602–609. 10.1128/AAC.00999-09 19995928 10.1128/AAC.00999-09 PMC2812160

[23] Karaca N, Şener G, Demirci B, Demirci F (2020) Synergistic antibacterial combination of *Lavandula latifolia* Medik. essential oil with camphor. Z Naturforsch C J Biosci 76(34):169–173. 10.1515/znc-2020-0051 33128531 10.1515/znc-2020-0051

[24] Manten A (1981) Side effects of antibiotics. Vet Q 3(4):179–182. 10.1080/01652176.1981.9693824 7292469 10.1080/01652176.1981.9693824

[25] Maertens JA (2004) History of the development of azole derivatives. Clin Microbiol Infect 10(1):1–10. 10.1111/j.1470-9465.2004.00841.x 14748798 10.1111/j.1470-9465.2004.00841.x

[26] Gupta D, Jain DK (2015) Chalcone derivatives as potential antifungal agents: Synthesis, and antifungal activity. J Adv Pharm Technol Res 6(3):114–117. 10.4103/2231-4040.161507 26317075 10.4103/2231-4040.161507 PMC4542397

[27] Belkaid Y, Hand TW (2014) Role of the microbiota in immunity and inflammation. Cell 157(1):121–141. 10.1016/j.cell.2014.03.011 24679531 10.1016/j.cell.2014.03.011 PMC4056765

[28] Hirsch C, Schildknecht S (2019) In vitro research reproducibility: keeping up high standards. Front Pharmacol 10:1484. 10.3389/fphar.2019.01484 31920667 10.3389/fphar.2019.01484 PMC6916005

[29] Ellis RJ (2022) Questionable research practices, low statistical power, and other obstacles to replicability: why preclinical neuroscience research would benefit from registered reports. eNeuro. 10.1523/ENEURO.0017-22.2022 35922130 10.1523/ENEURO.0017-22.2022 PMC9351632

[30] Xu M, Wu P, Shen F, Ji J, Rakesh KP (2019) Chalcone derivatives and their antibacterial activities: current development. Bioorg Chem 91:103133. 10.1016/j.bioorg.2019.103133 31374524 10.1016/j.bioorg.2019.103133

[31] Nematollahi MH, Mehrabani M, Hozhabri Y, Mirtajaddini M, Iravani S (2023) Antiviral and antimicrobial applications of chalcones and their derivatives: from nature to greener synthesis. Heliyon 9(10):e20428. 10.1016/j.heliyon.2023.e20428 37810815 10.1016/j.heliyon.2023.e20428 PMC10556610

[32] Mezgebe K, Melaku Y, Mulugeta E (2023) Synthesis and pharmacological activities of chalcone and its derivatives bearing N-heterocyclic scaffolds: a review. ACS Omega 8(22):19194–19211. 10.1021/acsomega.3c01035 37305270 10.1021/acsomega.3c01035 PMC10249103

[33] Stein C, Makarewicz O, Bohnert JA, Pfeifer Y, Kesselmeier M, Hagel S, Pletz MW (2015) Three-dimensional checkerboard synergy analysis of colistin, meropenem, tigecycline against multidrug-resistant clinical klebsiella pneumonia isolates. PLoS ONE 10(6):e0126479. 10.1371/journal.pone.0126479 26067824 10.1371/journal.pone.0126479 PMC4465894

[34] Ballan R, Saad SMI (2021) Characteristics of the gut microbiota and potential effects of probiotic supplements in individuals with type 2 diabetes mellitus. Foods 10(11):2528. 10.3390/foods10112528 34828808 10.3390/foods10112528 PMC8622611

