# Supplementary material for: Selective in vitro Synergistic Evaluation of Probiotic Tolerant morpholinyl- and 4-ethylpiperazinyl-Imidazole-chalcone Derivatives on Gastrointestinal System Pathogens
Source: Curr Microbiol. 2024 Jul 3;81(8):258. doi: 10.1007/s00284-024-03788-5 (PMC11222229; doi:10.1007/s00284-024-03788-5)
Supplement: Supplementary file 1 — Supplementary file1 (DOCX 212 kb) [file 284_2024_3788_MOESM1_ESM.docx]

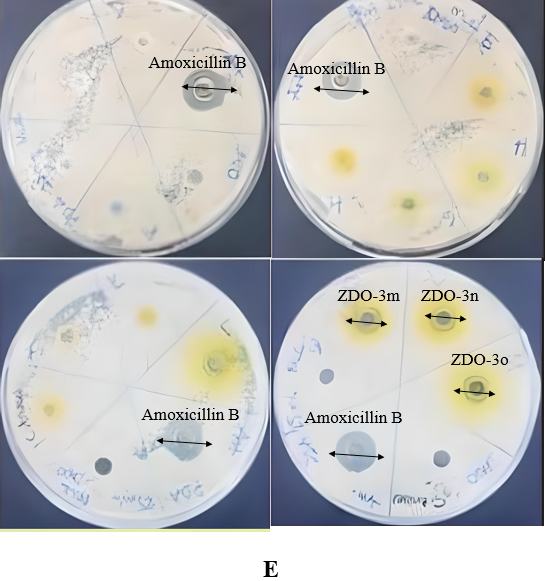

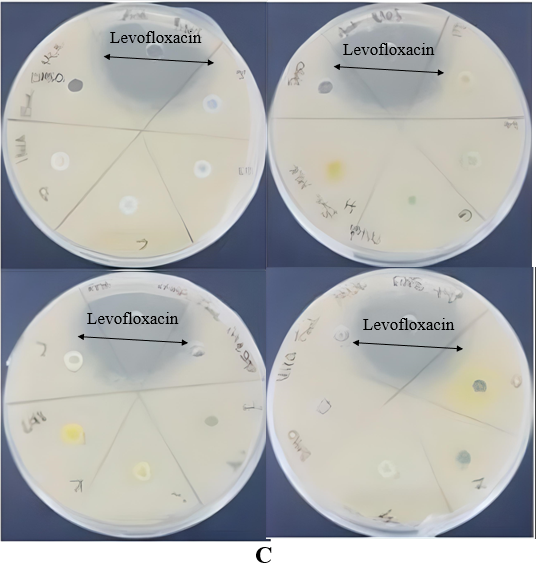

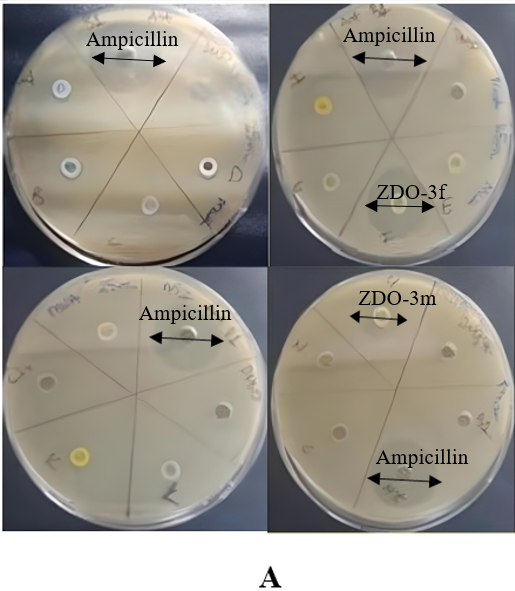

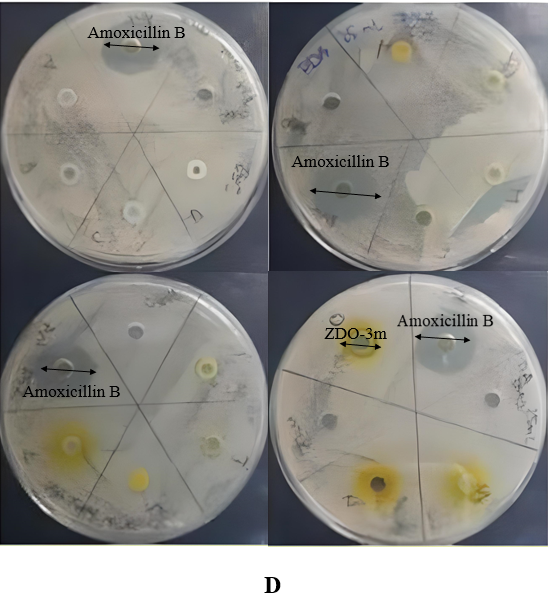

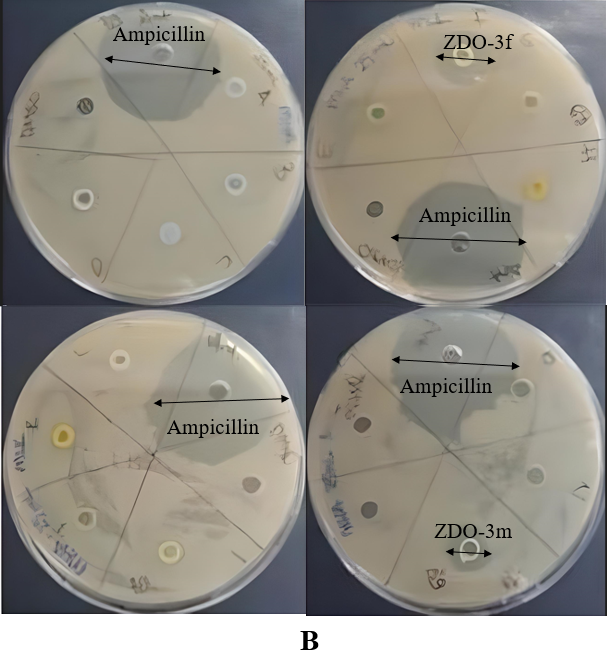


**Figure 2:** Pathogen Agar Well Diffusion Test

**A-** *E. coli* **B-** *B. subtilis* **C-** *C. Difficile* **D-** *C. albicans* **E-** *C. krusei*
