# Supplementary material for: Selective in vitro Synergistic Evaluation of Probiotic Tolerant morpholinyl- and 4-ethylpiperazinyl-Imidazole-chalcone Derivatives on Gastrointestinal System Pathogens
Source: Curr Microbiol. 2024 Jul 3;81(8):258. doi: 10.1007/s00284-024-03788-5 (PMC11222229; doi:10.1007/s00284-024-03788-5)
Supplement: Supplementary file 2 — Supplementary file2 (DOCX 1703 kb) [file 284_2024_3788_MOESM2_ESM.docx]

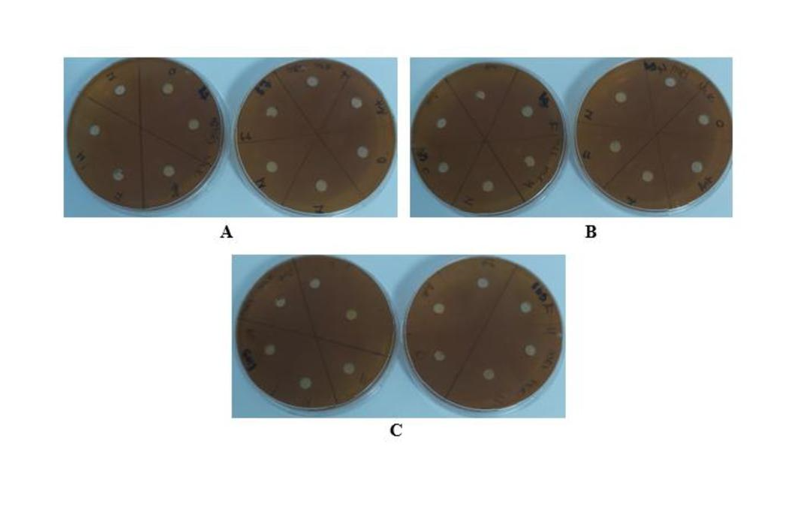


**Figure 1:** Probiotic Agar Well Diffusion Test

**A-** *Lactobacillus fermentum* (CECT-5716), **B-** *Lactobacillus rhamnosus* (GG), **C-** *Lactobacillus casei* (RSSK-591),
